# Longitudinal lung function and structural changes in children with primary ciliary dyskinesia

**DOI:** 10.1186/2046-2530-1-S1-P3

**Published:** 2012-11-16

**Authors:** S Blanchon, M Lémery Magnin, P Cros, E Escudier, M Mahloul, A Tamalet, A Clement, H Ducou Le Pointe, N Beydon

**Affiliations:** 1 Department of Pediatric Pulmonology, Hôpital Trousseau, France; 2 Department of Pediatric Radiology, Hôpital Trousseau, France; 3 Department of Medical Genetics and Embryology, Hôpital Trousseau, France; 4 INSERM, Research Unit 707, Hôpital Saint Antoine, France; 5 Lung Physiology, Hôpital Trousseau, France

## Background

Functional and structural lung evaluations are part of the follow-up of patients with primary ciliary dyskinesia (PCD). We aimed to evaluate transversal and longitudinal relationships between lung function tests (LFT) and chest computed tomography (CT) in children with PCD, in stable clinical condition.

## Methods

Data from children followed in the French National Center were retrospectively collected. Inclusion criteria were (i) definitive diagnosis of PCD, (ii) age less than 15 years at the beginning of follow-up, (iii) at least 8 years of follow-up and (iv) at least 2 couples of concurrent CT and LFT performed in stable clinical condition. Twenty children (median age 4.6 years, median follow-up 15.4 years) were included. Concurrent LFT (blood gas and spirometry) and CT (score) results were recorded.

## Results

LFT indices (PaO2 (n=210), FVC, FEV1, FEF2575% (n=195)) significantly decreased with age, and the mean annual decrease (z-score (% predicted)) was -0.17 (-0.49%), -0.09 (-0.50%), -0.10 (-0.89%), and -0.07 (-1.73%), respectively. First CT (median age 8.7 years) revealed bronchiectasis (70%), mucous plugging (70%), peribronchial thickening (90%), parenchymal abnormalities (65%) and hyperinflation (50%). CT-scores (n=74) significantly increased with age, and was negatively correlated to PaO2, FVC, FEV1 and FEF2575% longitudinal changes.

## Conclusion

In stable clinical condition, functional and structural progressive impairments significantly correlated in children with PCD. Further prospective studies, including large populations with various levels of disease severity, are needed to confirm whether LFT follow-up can be used to adjust CT frequency and help at minimizing the radiation burden in children with a good life expectancy.

